# Functional *IL-23R* rs10889677 Genetic Polymorphism and Risk of Multiple Solid Tumors: a Meta-Analysis

**DOI:** 10.1371/journal.pone.0080627

**Published:** 2013-11-20

**Authors:** Shanliang Zhou, Yueqin Ruan, Hongchen Yu, Yunzhi Chen, Yongjun Yao, Yanhui Ma, Yan Gao

**Affiliations:** 1 Clinical Laboratory, Cancer Hospital of Shandong Linyi, Shandong Linyi, China; 2 Clinical Laboratory, Affiliated Hospital of Binzhou Medical College, Shandong Binzhou, China; 3 Qingdao Orthopedics and Traumatology Hospital, Shandong Qingdao, China; 4 Clinical Laboratory, People's Hospital of Chengwu County, Shandong Heze, China; 5 Department of Pathology, People’s Hospital of Cangshan County, Shandong Linyi, China; 6 Qilu Children's Hospital of Shandong University, Jinan, China; Nanjing Medical University, China

## Abstract

Interleukin-23 receptor (IL23R) can interact with IL-23 and, thus, is involved in the T-helper 17 (Th17) cell-mediated inflammatory process as well as tumorigenesis. Recently, a functional single nucleotide polymorphism (SNP) rs10889677 has been identified in the 3’-untranslated region of *IL-23R*. It has been showed that the rs10889677AC SNP could increase the binding affinity of microRNA let-7f and downregulate IL-23R expression. Several case-control studies have examined the association between this SNP and genetic susceptibility of multiple solid tumors. However, the conclusions are conflicting. Therefore, we conducted this meta-analysis to systematically study the role of this functional *IL-23R* SNP in development of multiple solid tumors. There are a total of 5 studies are eligible (6731 cases and 7296 healthy controls). Either fixed-effect model or random-effect model was used to calculate pooled odds ratios (ORs) and the 95% confidence interval (95% CI). Significant association between this functional rs10889677 genetic variant and risk of multiple solid tumors were observed (CC genotype vs. AA genotype: OR = 0.59, 95% CI = 0.53-0.66, *P* < 0.001). These findings demonstrated that the *IL-23R* rs10889677 genetic variant might play an important part during malignant transformation of multiple solid tumors.

## Introduction

Interleukin-23 receptor (IL23R) can interact with IL-23 and, thus, is involved in the T-helper 17 (Th17) cell-mediated inflammatory process as well as tumorigenesis [1,2]. Th17 cells belongs to pro-inflammatory CD4^+^ effecter T-cells and can mediate tissue inflammation by secreting high levels of the pro-inflammatory cytokine IL-17 in response to stimulation [3]. IL-23R is involved in multiple important biological processes, including Th17 cell-mediated immune response, tumor-promoting pro-inflammatory processes and the failure of the adaptive immune surveillance [1,2]. IL-23R could lessen immunosurveillance by CD8^+^ T cells and accelerate tumor proliferation as well [4]. For regulatory T cells (Tregs), the IL-23R signaling pathway might also promote the immunosuppressive function of Tregs facilitating evasion of the immune system by cancer cells [5-7]. Interestingly, both Th17 cells and Tregs can enhance proliferation of cancer cells [8]. These findings suggest that IL-23R might play an important part during carcinogenesis and progression.

Several single nucleotide polymorphisms (SNPs) have been identified in *IL-23R* locating on chromosome 1p31.3. Notably, there is an rs10889677AC SNP in the 3’-untranslated region (3’-UTR) of *IL-23R*, which could increase the binding affinity of microRNA let-7f and, thus, decreased *IL-23R* transcription *ex vivo* and *in vivo*. Biological studies indicated that rs10889677CC carriers had less Tregs and a faster T-cell proliferation than individuals carrying the rs10889677AA homozygous genotype [9]. Several case-controls studies have examined the association between rs10889677 SNP and risk of multiple solid tumors, such as breast cancer, lung cancer, ovarian cancer, gastric cancer, nasopharyngeal carcinoma, and oral cancer [9-13]. However, the results are inconsistent and inconclusive. Due to the importance of IL-23R during tumorigenesis, we systematically investigated the role of functional *IL-23R* rs10889677 genetic variant on multiple solid tumors through a meta-analysis.

## Materials and Methods

### Literature search and data extraction

The electronic literature searches were done with search terms of “Interleukin-23 receptor”, “IL-23 receptor”, “IL23R”, “polymorphism”, “variant”, “SNP”, “rs10889677”, “cancer”, “tumor”, as well as their combinations using HuGE Navigator (version 2.0), PubMed (US National Library of Medicine, National Institutes of Health), EMBASE and Web of science [14-16]. Case-control studies of the *IL-23R* rs10889677 SNP published from October, 2009 to June, 2013 were identified without language restrictions. Additional studies have also identified by screening reference lists of important studies and reviews. Criteria for selecting an eligible study: (i) original studies; (ii) studies that investigated the association between *IL-23R* rs10889677 polymorphism and solid tumor risk; (iii) studies that reported crude odds ratio (OR) with 95% confidence interval (CI) values or sufficient data to calculate crude OR and 95% CI. Criteria for exclusion of studies were (i) overlapping data and (ii) case-only studies, family-based studies and review articles. The raw data and demographic information, including first author, published year, population, sample size, cancer types and genotypes were independently extracted. 

### Statistical analysis

Association between *IL-23R* rs10889677 SNP and solid tumor risk were re-calculated using crude ORs together with their corresponding 95% CIs. If the P value of the heterogeneity test was ≥ 0.05, we used the fixed effect model to calculate the combined OR (the Mantel-Haenszel method) [17]. The fixed effect model assumed the same homogeneity of effect size across all studies. If the P value of the heterogeneity test was <0.05, there is statistically significant between-study heterogeneity. Then, we would use a random effects mode (the DerSimonian and Laird method) to calculate the combined OR [18]. Funnels plots was utilized to test publication bias. Egger’s test was used to evaluate the funnel plot asymmetry [19], which can indicate potential existing publication bias. The statistical analyses were performed using Stata Statistical package (version 11.0; Stata Corp., College Station, Tex). All *P* values were two-sided. Statistical power in relation to the minor allele frequencies (MAFs) of the *IL-23R* rs10889677 SNP were calculated with Quanto 1.2.4 software [20].

## Results

### Literature search and data extraction

After searching HuGE Navigator, NCBI PubMed , EMBASE and Web of science using the keywords “Interleukin-23 receptor”, “IL-23 receptor”, “IL23R”, “polymorphism”, “variant”, “SNP”, “rs10889677”, “cancer”, or “tumor”, and found 5 studies (6731 cases and 7296 healthy controls), which fulfilled inclusion criteria [9-13]. There are two studies on breast cancer [9,12], one on lung cancer [9], one ovarian cancer [10], one on gastric cancer [11], one on nasopharyngeal carcinoma [9], and one on oral cancer [13]. A database of genotype frequency and other information extracted from each study, was created. Table 1 showed the essential information, including first author, year of publication, SNPs genotyped, sample size,population, cancer types and genotyping methods. 

**Table 1:** Studies included in the meta-analyses of association between *IL-23R* rs10889677 SNP and multiple solid tumors.

No	Studies	Cases	Controls	Populations	Cancer types	Genotyping method
1	Zhang et al. 2010	96	115	Chinese	ovarian cancer	PCR-RFLP
2	Chen et al. 2011	962	787	Chinese	gastric cancer	PCR-RFLP
3	Zheng et al. 2012	1010	1014	Chinese	breast, lung and nasopharyngeal cancers	MALDI-TOF MS
4	Wang et al. 2012	491	502	Chinese	breast cancer	SNaP shot
5	Chien et al. 2012	240	240	Chinese	oral cancer	PCR-RFLP

Abbreviations: SNP, single nucleotide polymorphism; MALDI-TOF MS: Matrix-Assisted Laser Desorption/ Ionization Time of Flight Mass Spectrometry; PCR-RFLP: PCR-Restriction Fragment Length Polymorphism.

### Quantitative data synthesis

**Figure 1:**
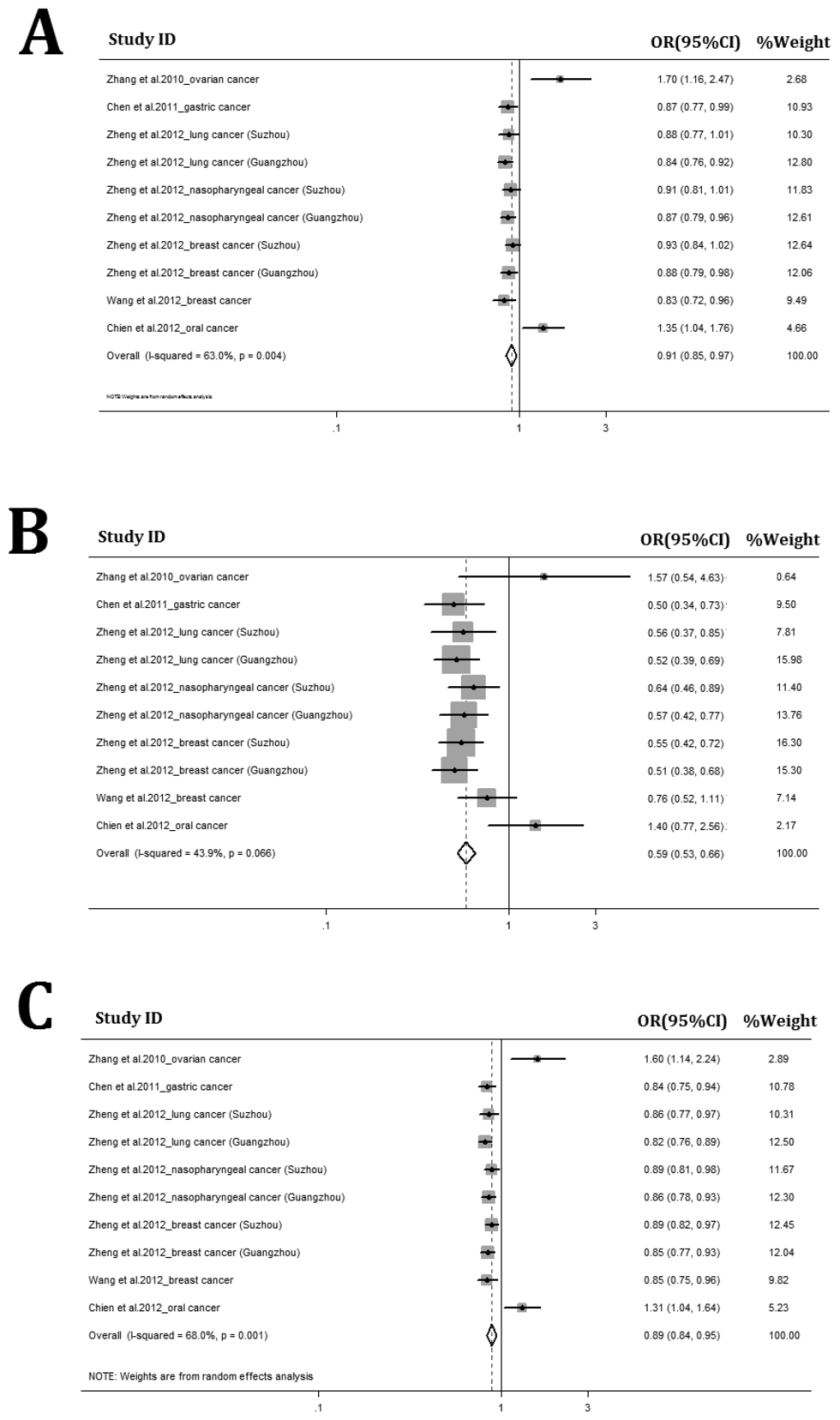
Meta-analyses for functional *IL-23R* rs10889677 polymorphism in multiple solid tumors. Forest plots for AC vs. AA comparison (A, CC vs. AA comparison (B), and AC + CC vs. AA comparison (C).

The association between the functional *IL-23R* rs10889677 SNP and risk of multiple solid tumors was investigated in 6731 cases and 7296 controls. Compared with the rs10889677 AA genotype, a 0.91-fold decreased risk to develop multiple solid tumors was observed for individuals with AC genotype in a random effect model (95% CI = 0.85-0.97, *P* = 0.006) (Figure 1A). The rs10889677 CC genotype was also associated with significantly decreased risk of multiple solid tumors compared to those AA genotype carriers (OR = 0.59, 95% CI = 0.53-0.66, *P* < 0.001) (Figure 1B). Interestingly, when we combined data of AC and CC genotypes, an OR of 0.89 (95%CI = 0.84-0.95, *P* < 0.001) was observed (Figure 1C). 

For the rs10889677 polymorphism with MAF equaling to 0.316 in healthy individuals, there is 94.6% or 99.9% statistical power to detect a 0.91-fold or 0.59-fold increased risk developing multiple solid tumors.

### Bias diagnostics

**Figure 2:**
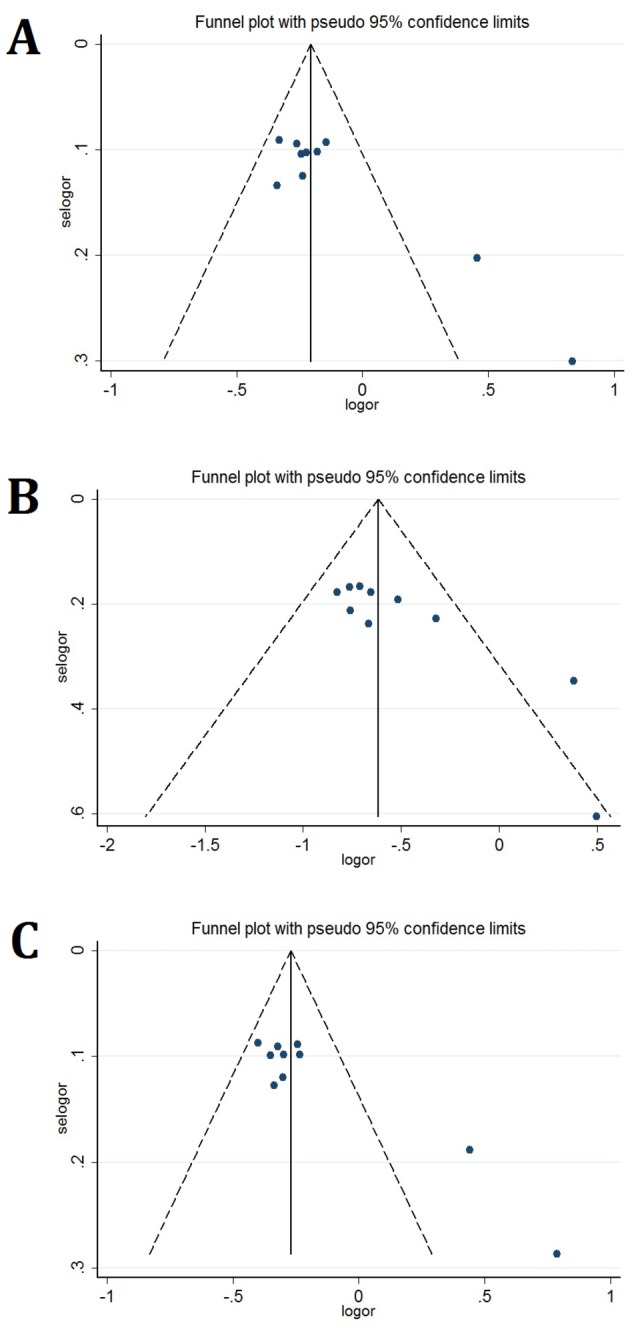
Funnel plots of the Egger’s test of *IL-23R* rs10889677 allele comparison for publication bias. Funnel plots for AC vs. AA comparison (A, CC vs. AA comparison (B), and AC + CC vs. AA comparison (C).

To evaluate publication bias, genotypes of *IL-23R* rs10889677 polymorphism were plotted against the precision ones using a funnel plot. However, we did observe that there was publication bias on studies of *IL-23R* rs10889677 polymorphism (Egger’s test: *P* < 0.05 (Figure 2).

## Discussion

IL-23R is essential for initiating, maintaining and accelerating the IL-23/IL-17 inflammatory signal transduction pathway, which is important during malignant transformation [1,2]. A functional rs10889677AC SNP has been associated with genetic susceptibility of solid tumors, including breast cancer, lung cancer, ovarian cancer, gastric cancer, nasopharyngeal carcinoma, and oral cancer [9-13]. However, no conclusions on how the genetic variant influences cancer risk have been made. The current meta-analysis including five literatures (6731 cases and 7296 healthy controls) systematically examined association between the rs10889677AC SNP and multiple cancer risk. We found that rs10889677 SNP might be common genetic susceptible factor for multiple solid tumors. 

Zheng et al demonstrated that *IL-23R* rs10889677 genetic variant is a regulatory genetic variant of *IL-23R* gene expression [9]. MiRNA let-7f can down-regulate *IL-23R* gene expression by binding to 3’UTR of the *IL-23R* mRNA and inhibit translation of IL23R protein. Interestingly, the rs10889677 A>C change locates in the binding sequence of let-7f, which could interfere let-7f-mediated repression of *IL-23R* expression. It has also been showed that there was higher expression level of IL-23R in peripheral blood mononuclear cells (PBMCs) from healthy individuals with rs10889677AA genotype than that among subjects with rs10889677AC or CC genotype. The *IL-23R* rs10889677 A allele carriers had more Tregs *in vivo* and lower T-cells proliferation rate *in vitro* compared to those with rs10889677 C allele [9]. All these findings might explain the observed decrease in cancer susceptibility in the meta-analysis of *IL-23R* rs10889677 SNP.

In all, we for the first time examined the involvement of functional *IL-23R* rs10889677 polymorphism during malignant transformation of multiple solid tumors through a systematic way. Our findings demonstrated that genetic variants that impact the efficacy of the immune system may modify cancer risk [21-23].

## Supporting Information

Checklist S1 **PRISMA checklist.**
(DOC)
